# A brief history of the development and use of vulcanised rubber in dentistry

**DOI:** 10.1038/s41415-023-5735-7

**Published:** 2023-04-28

**Authors:** Christopher Stephens

**Affiliations:** Emeritus Professor of Child Dental Health, University of Bristol, Bristol, United Kingdom

## Abstract

Until the late nineteenth century, denture baseplates and removable orthodontic appliances were usually made of gold. Though now largely forgotten, the discovery of vulcanised rubber was instrumental in making these forms of dental treatment widely available to the general public. While Charles Goodyear is recognised for his role in the development of vulcanite in America, that of Thomas Hancock in the UK is less well-known.

## Key points

- Describes the discovery of latex rubber (also then known as caoutchouc or elastic gum) in the late eighteenth century and how this material came to be known to the western world.
- Describes the parallel development of vulcanised rubber in the nineteenth century by Thomas Hancock in England and Charles Goodyear in America.
- Suggests that the popularity of removable orthodontic appliances made from vulcanite in nineteenth century America was affected by the patents held by the Goodyear Company, which limited the dental use of the new material in that country.

## The origins of rubber

The raw material for rubber comes from the sap of the rubber tree (*Castilla elastica*), which is indigenous to the tropical areas of Southern Mexico and Central America, but is now grown widely in South East Asia. The first known use of the tapped latex, then also known as caoutchouc or elastic gum, was by the pre-Columbian civilisations of Central America, where it was used to make balls for a game, of which ulama is the modern descendant.

François Fresneau (1703-1770) a French engineer, botanist and explorer, is credited with having written the first scientific paper on rubber. Aged 29 years, Fresneau had been sent to French Guiana to design the fortifications of the colony's capital, Cayenne, and to determine the natural resources of the colony. Returning to France, he suggested that natural rubber (that is, latex) might be used as waterproofing material.^1^ In England in 1770, the chemist Joseph Priestley observed that a piece of the new material was extremely good for rubbing out pencil marks on paper; hence, it was soon known in England as 'rubber'.

## The development of vulcanite in England and the United States

Untreated latex consists of polymers of the organic compound isoprene, with minor impurities of other organic compounds. Vulcanisation is the process by which the latex is heated with sulphur to create molecular crosslinks between the long rubber molecules. Increasing the sulphur content increases its hardness and tensile strength. Adding between 25-50% sulphur produces a brown material with the feel of plastic. The discovery of the process of vulcanisation of rubber is associated with the names of Hancock in England and Goodyear in America.

**Fig. 1:**
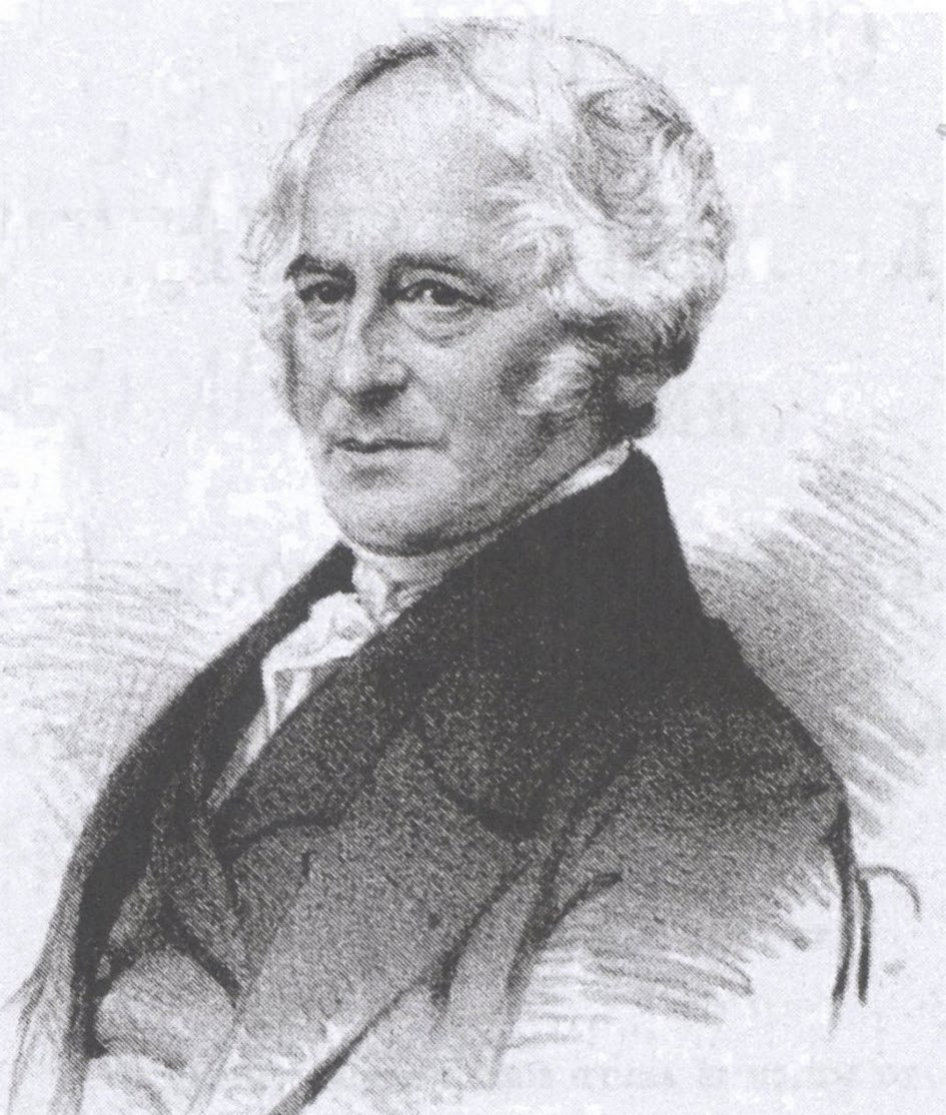
Thomas Hancock (1786-1865). Image reproduced from T. Hancock, *The origin and progress of the Caoutchouc, or India-Rubber Manufacture in England*, London: Longmans & Roberts, 1857^3^

**Fig. 2:**
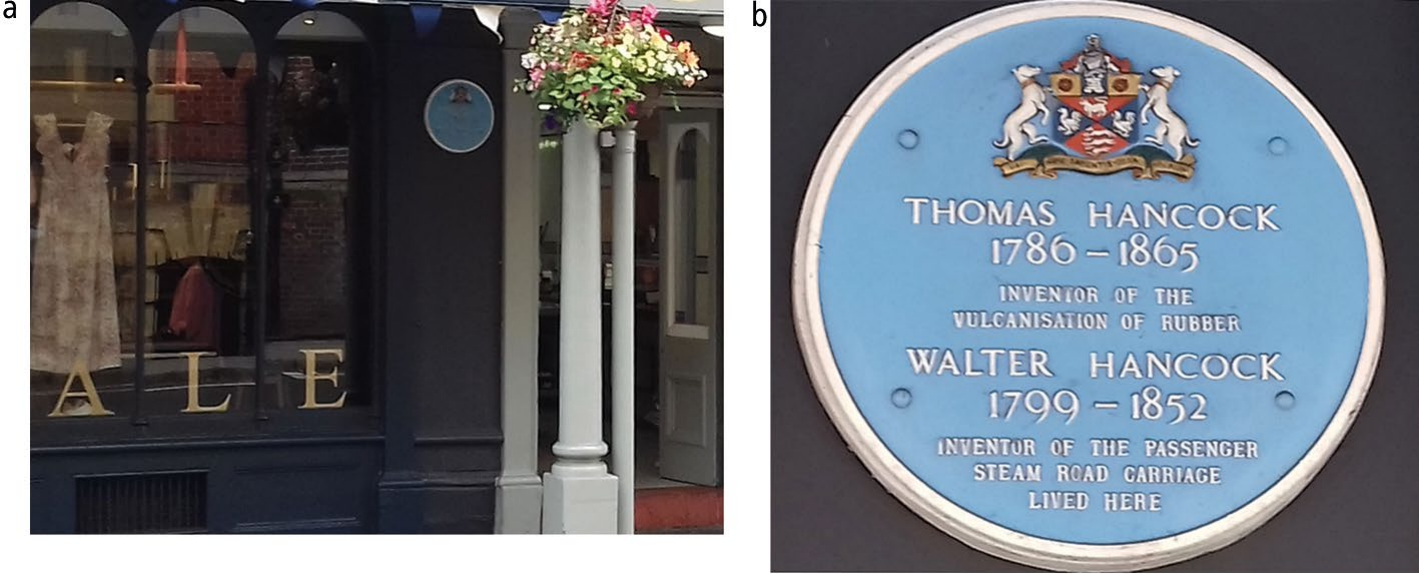
a, b) Oxford Street, Marlborough, Wiltshire; the birthplace of Thomas Hancock

Thomas Hancock (1786-1865) was one of six brothers, born in Marlborough, Wiltshire (Fig. 1, Fig. 2).^2^ In 1815, he is recorded as being in partnership with his younger brother, Walter, who was an engineer based in London. Thomas' interest in rubber seems to have been in developing a material suitable for waterproofing his brother's carriages. He discovered that latex combined with pitch and tar could be used for this purpose and could also be applied to canvas, rope and other fibrous material, for which he took out several patents starting in 1820.^3^ It is of note that by 1824, his brother Walter had produced a workable steam carriage engine, in which the pistons were replaced with two flexible bags made of layers of canvas united by 'India rubber solution'.^4^

In 1826, Thomas Hancock presented his findings on latex to the Royal Institution in London, as a result of which Michael Faraday, the most influential scientist of his era, published a report on its chemistry.^5^ Having developed a process for making India rubber sheeting in 1837, Hancock became a partner in the firm of Charles Macintosh and Co (after whom the waterproof macintosh is now known), but he also still carried on his own business in London.^6^ There followed a rapid expansion in the number of rubber products produced by the partners, including soles for shoes, bellows, cushions, inflatable life belts and pontoons.

Hancock never claimed to have been the inventor of the vulcanisation process. In the Autumn of 1842, his friend and inventor William Brockendon FRS (Fellowship of the Royal Society) had shown him pieces of rubber which had been hardened by a secret process. These were American samples provided by an agent of its American inventor. This may well have been Charles Goodyear himself, who is known to have visited Britain at this time.

Charles Goodyear (1800-1860) was 33 years old when he decided to venture into rubber products. This was after his father's New Haven hardware business went bankrupt. During the next ten years, Goodyear became obsessed with the development of what he called 'gum elastic', borrowing money to pursue his experiments.^7^ According to his later biographers, it was while working at the Eagle India Rubber Company in Woburn, Massachusetts in 1839 that Goodyear accidentally combined rubber and sulphur upon a hot stove. Much to his surprise, the rubber didn't melt and, when he raised the temperature further, it actually hardened. Thirty years after Goodyear's death, numerous rubber companies had been established in his home town of Naugatuck, Connecticut and in 1892, nine of these merged to form The United States Rubber Company (now Uniroyal).

During their 1842 meeting, Hancock and Brockendon advised the American agent to tell his employer to take out a patent for the new product, but they also noted that his samples smelt of sulphur. This meeting appears to have redirected Hancock's own research and resulted in him patenting his own process in November 1843, which Brockendon was later to call vulcanisation, after the Roman god of fire. This was eight weeks before Charles Goodyear filed his own patent in the USA, thereby blocking the latter's attempt to secure a British patent. By 1847, Hancock had taken out 16 British patents for variations in the vulcanising process, among which was the discovery that if the process was continued at a higher temperature, a hard substance (known as ebonite) was produced, which was impervious to chemical attack and was an excellent insulator, which made it of considerable worth to the emerging electrical industry.

## Vulcanised rubber and dentistry

Until the mid-nineteenth century, gold was the only satisfactory baseplate material for dentures and removable orthodontic appliances.^8^^,^^9^ In 1835, Goodyear had approach C. S. Putnam, a New York dentist, with the suggestion that the material might have a dental application. But it was the American dentist, Thomas Witberger Evans (1823-1897), who claimed to be the first to use rubber as a denture baseplate material.^10^ This was in 1848, while he was working as surgeon-dentist to Louis-Napoleon, the future Napoleon III of France, from whom he received the *Legion d'Honneur.* Curiously, despite serving the Emperor in this capacity for 50 years, there is no mention of this claim in Evan's extensive and detailed memoire.^11^ (When Evans died in 1897, he left his fortune for the creation a dental school in his native Philadelphia, which has now become the University of Pennsylvania School of Dental Medicine).

Both Goodyear, Macintosh and Hancock exhibited rubber products in London at the Great Exhibition of 1851, which had been promoted by Prince Albert after the success of the Paris Exposition of 1849. However, only Macintosh advertised his product as vulcanised rubber and there is no mention of a dental use of the new material in the official catalogue*.*^12^ He later received one the organising council's medals for outstanding achievements, which were awarded to 170 of the 15,000 exhibitors.

Macintosh and Hancock's stands were located in the North Gallery of the Crystal Palace as part of the Exhibition's Class 28 - 'Manufactures from animal and vegetable substances not being woven or felted'. Goodyear's stand was included in the section for 'Other world nations'. Almost certainly these exhibitors met in the course of the six-month exhibition, which finally closed in October 1851, but there is no record of them doing so.

In the following year, Goodyear visited Paris (and maybe London as well). Thomas Evans, having heard of Goodyear's success in stabilising vulcanised rubber, called on him there and discussed its dental uses.^13^ Back in New York, by 1856, Putnam had succeeded in overcoming the problem of pressure vulcanising. In the same year, he published his own book, but in it only describes the use of gutta percha as a denture base material.^14^ In 1858, Goodyear took out a new patent, which covered the process for the dental use of vulcanite. Evans, learning of this, was greatly offended, as he believed the discovery should have been made freely available to dental science. It would seem that as a result of this, he published a leaflet claiming that he had been the first to make a wearable denture in the new material.^10^ Another contender for this claim was Dr. John A. Cummings of Boston. He submitted his patent notice in 1852 and completed his application in 1855. Initially, the American patent office refused Cummings' application and it was not until 1864 that his patent was finally issued (patent number 43,009). This was the same year in which Charles Goodyear founded the Goodyear Dental Vulcanite Company. As Goodyear gave half the rights for the dental use of vulcanite in New York to Putnam, this strongly suggests there was an agreement between Goodyear and Putnam on the scope of his book, which was perhaps to establish Putnam as an authority on new materials while the new patent for vulcanite was being obtained.^15^

Meanwhile, in 1844, Samuel Stockton White (1822-1879), a dentist from Philadelphia, had founded the S. S. White Dental Company and began publishing the *Dental Cosmos* which ran from 1859-1936. In 1865, he published details of the vulcanising process for dentistry and soon, many thousands of American dentists were making vulcanite dentures.^16^ This was hardly surprising in view of huge cost savings, which meant that for the first time, dentures could be afforded by the middle classes. The growth in demand was also stimulated by the discovery of the first effective local anaesthetic by Jayne in 1841.^17^ The Goodyear Company, having by this time acquired the Cumming's patent in addition to their own, now claimed that every dentist needed to have a $45 Goodyear licence to use the new material for dental purposes. There followed a legal battle with the dental profession, which lasted until the Supreme Court ruling of October 1876.^18^^,^^19^ In the interim, Goodyear's financial director, Josiah Bacon, single-mindedly set out on a 13-year campaign to track down and prosecute non-licenced dentists. The Company's struggle with the profession reached a climax in 1879, when Bacon was shot dead in a Californian hotel by the irate dentist Samuel Chalfant.^7^ The Goodyear patents finally expired in 1881, at which point vulcanite came into general dental use world-wide.

**Fig. 3:**
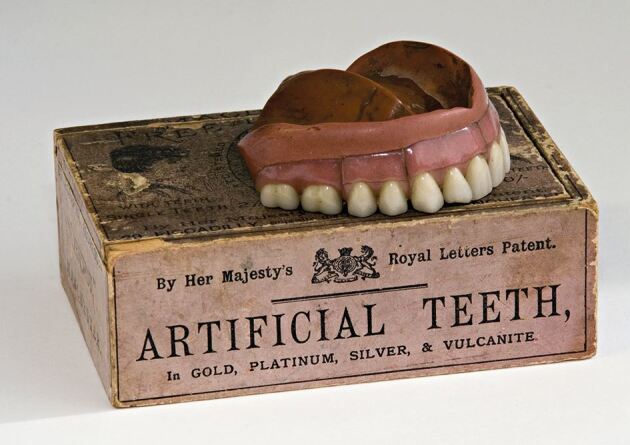
Showing the use of porcelain sections to improve the appearance of a vulcanite denture. Image reproduced with kind permission from the BDA Museum

**Fig. 4:**
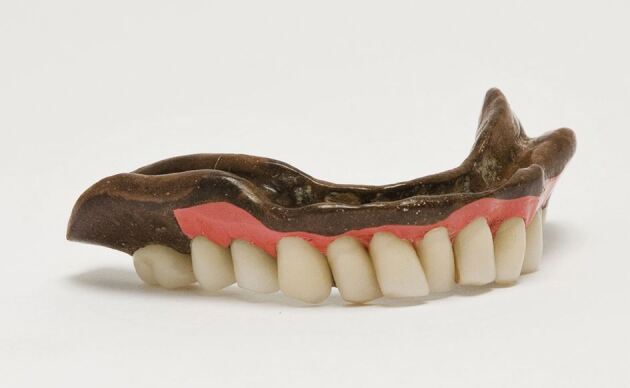
Lower denture in which the tooth bearing sections only have been made in red vulcanite. Image reproduced with kind permission from the BDA Museum

In England, Hancock had imposed no such restriction in the dental use of his patent and by 1857, vulcanised rubber had already found an application in appliances for the treatment of cleft palate.^20^ Its adoption as a baseplate material for dentures and orthodontic appliances soon followed, where, in an effort to improve the material's colour, up to 30%. vermillion (mercuric sulphide [HgS]) was soon being added by dental supply companies (Fig. 3, Fig. 4). Concern was expressed on both sides of the Atlantic that the vermillion colouring could cause poisoning.^21^ However, the Committee appointed by the Odontological Society of Great Britain in 1876 to investigate this suggestion failed to establish a single case of even probable mercurial poisoning due to red vulcanite, and no further action was taken.^22^

## Vulcanite in orthodontics

**Fig. 5:**
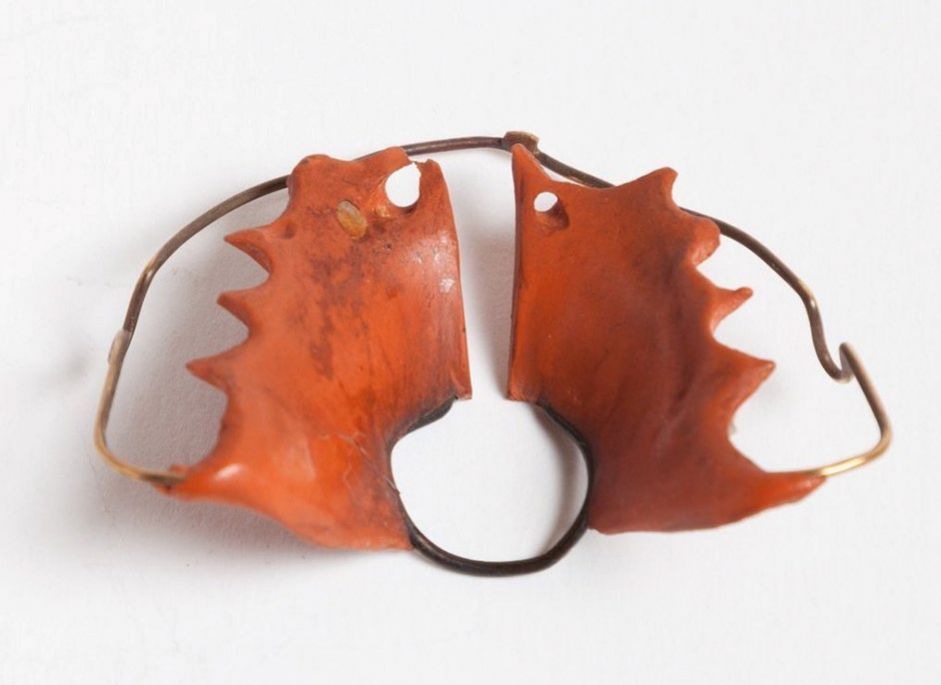
Showing a typical red vulcanite orthodontic expansion appliance. Image reproduced with kind permission from the BOS Museum

It may be that the ongoing legal dispute in America discouraged American orthodontists from employing removable appliances made from vulcanite, which were soon adopted in England and Europe. By 1876, Charles Gaine of Bath was using vulcanite instead of gold for his orthodontic appliances.^23^ In the same year, J. S Turner, who had succeeded Robert Hepburn as Lecturer in Dental Surgery and Mechanics at the Dental Hospital of London, was including in his teaching course: 'Vulcanite its nature and preparation. Making vulcanite cases. Making pivots. Mounting spiral springs. Regulation plates'.^24^ By the turn of the century, vulcanite orthodontic appliances were the norm,^25^ but a few dental practices in West London were slower to adopt the new material. Even as late as 1915, Norman Bennett, President of the British Society for the Study of Orthodontics, commenting on a paper presented to the Society, stated that *'*he [had] preferred gold plates to vulcanite, but found he was using more vulcanite plates now than gold ones...there was much to be said for the vulcanite plate, which was certainly a more snugly fitting plate than one of cast metal'.^26^ The latter comment appears to imply that the slight elasticity of the vulcanite and the palatal undercuts of the upper posterior teeth aided the retention of a maxillary orthodontic appliance and, as suggested by Trenouth,^27^ explains why many upper arch expansion plates were made in vulcanite without any form of clasping (Fig. 5).

## The end of the vulcanite era

Polymethyl methacrylate (acrylic) was developed in the early 1930s by the British chemists Rowland Hill and John Crawford of Imperial Chemical Industries, which then manufactured various forms of the product at their Billingham factory under the tradenames of Diakon, Perspex and Kallodent, and would later produce and sell their own curing oven.^28^ Acrylic only became available in the UK at the end of 1941.^29^ Initially, Kallodent was supplied as a filling material, but was made available for dental research purposes.^30^Thus, its use soon spread from dental conservation to prosthetics.^31^^,^^32^

The adoption of acrylic by the orthodontic speciality is less well-documented, but in the UK, the illustrations in the 1947 *Transactions of the British Society for the Study of Orthodontics*^33^clearly show that by this time, most, if not all removable appliances were being made in the new material. It seems that elsewhere in Europe this changeover may not have been quite so rapid, as an abstracted Dutch report of 1949 stated that the removable orthodontic appliance described could be made in either vulcanite or acrylic.^34^ It is perhaps surprising that, given the slow acceptance of vulcanite in the nineteenth century, the adoption of acrylic in Britain should have been so rapid, but it needs to be remembered that Japan's conquest of the Dutch East Indies in 1942 eliminated 90% of the world's sources of rubber.^35^This stimulated the rapid development and deployment of alternative materials throughout the West and also created a synthetic rubber industry in America.
